# Polarization Dependence on the Optical Emission in Nd-Doped Bioactive W-TCP Coatings

**DOI:** 10.3390/jfb17010038

**Published:** 2026-01-13

**Authors:** Daniel Sola, Eloy Chueca, José Ignacio Peña

**Affiliations:** 1 Aragonese Foundation for Research and Development (ARAID), 50018 Zaragoza, Spain; 2 Instituto de Nanociencia y Materiales de Aragón, Universidad de Zaragoza—CSIC, 50018 Zaragoza, Spain; dumpty_heavenly@hotmail.es (E.C.); jipena@unizar.es (J.I.P.)

**Keywords:** optical properties, luminescence, rare-earth-doped materials, laser floating zone, directionally solidified ceramic eutectics, bioceramics

## Abstract

Neodymium-doped bioactive wollastonite–tricalcium phosphate (W-TCP:Nd) coatings were fabricated by combining dip-coating and laser floating zone (LFZ) techniques to investigate the dependence of optical emission on polarization. Structural and spectroscopic analyses were performed on both longitudinal and transversal sections of the coating to assess the effects of directional solidification on luminescence and vibrational behavior. Micro-Raman spectroscopy revealed that the coating exhibited sharp, well-defined peaks compared to the W-TCP:Nd glass, confirming its glass-ceramic nature. New Raman modes appeared in the longitudinal section, accompanied by red and blue shifts in some bands relative to the transversal section, suggesting the presence of anisotropic stress and orientation-dependent crystal growth. Optical emission measurements showed that while the ^4^F_3/2_→^4^I_11/2_ transition near 1060 nm was nearly polarization independent, the ^4^F_3/2_→^4^I_9/2_ transition around 870–900 nm exhibited strong polarization dependence with notable Stark splitting. The relative intensity and spectral position of the Stark components varied systematically with the rotation of the emission polarization. These findings demonstrate that directional solidification induces polarization-dependent optical behavior, indicating potential applications for polarization-sensitive optical tracking and sensing in bioactive implant coatings.

## 1. Introduction

Bioceramics play a fundamental role in modern medicine due to their excellent biocompatibility, durability, and ability to interact positively with biological tissues. They are widely used in applications such as bone grafts, dental implants, and joint replacements, where their similarity to natural bone structure promotes healing and tissue integration [1]. Unlike traditional materials, bioceramics can be engineered to be bioinert, bioactive, or even biodegradable, depending on the medical need, making them highly versatile. Their resistance to wear and corrosion also ensures long-term performance in the body, improving patients’ quality of life and reducing the need for repeated surgeries [2]. Therefore, bioceramics are becoming increasingly important in regenerative medicine and drug delivery systems due to their potential applications in healthcare.

Both wollastonite (W) and tricalcium phosphate (TCP) are important bioceramics that have gained significant attention in biomedical applications because of their excellent bioactivity and ability to support bone regeneration [3,4,5]. Tricalcium phosphate promotes strong bonding with natural bone by forming a hydroxyapatite layer on its surface when in contact with body fluids, thereby enhancing osteoconductivity. Wollastonite, on the other hand, is highly biocompatible and biodegradable, gradually dissolving in the body while stimulating new bone growth, making it ideal for bone grafts and scaffolds [3,4,5,6,7]. When used individually or in composites, these bioceramics not only provide structural support but also actively participate in the healing process, accelerating bone repair and reducing recovery time. Their combined properties are essential for the development of advanced biomaterials for orthopedic and dental implants [8].

Adding rare-earth ions to bioceramics like wollastonite and tricalcium phosphate can significantly enhance their biological and functional properties, making them more effective for medical purposes [9,10,11]. Rare-earth ions such as cerium, europium, or gadolinium can improve bioactivity by stimulating cell proliferation, differentiation, and bone tissue formation [12,13,14]. Some of these ions also introduce antibacterial properties, helping to reduce the risk of post-surgical infections [15,16]. In addition, certain rare-earth ions provide luminescent or magnetic characteristics. The study of luminescence in bioceramics enables the combination of structural and therapeutic functions with advanced diagnostic capabilities. Incorporating luminescent elements into bioceramics allows, under specific light excitation, real-time tracking of implants and monitoring of their integration with bone tissue without invasive procedures, which is especially valuable for bioimaging [17,18,19,20]. Luminescence can also provide insights into the material’s microstructural properties, which directly affect its biological performance. In particular, the assessment of the optical polarization of luminescence in bioceramics provides information on the symmetry, orientation, and local environment of luminescent ions within the ceramic matrix [21,22]. By analyzing polarization, it is possible to determine how crystal structures, defects, and ion substitutions influence light emission, which in turn affects the efficiency and stability of luminescent properties. This knowledge is particularly valuable for designing bioceramics with controlled optical responses. Among rare-earth ions, Nd^3+^ is particularly suitable for this study because it exhibits well-defined and Stark-resolved emission manifolds in the near-infrared region [23,24]. These characteristics make Nd^3+^ an excellent spectroscopic probe of local crystal-field symmetry, microstructural orientation, and polarization-dependent optical behavior. In addition, Nd^3+^ has been widely incorporated into oxide and bioactive ceramic hosts, ensuring optical stability and allowing direct comparison with previous spectroscopic studies [24].

In this work, neodymium-doped wollastonite–tricalcium phosphate (W–TCP:Nd) coatings were fabricated on the surface of calcium zirconate-calcium-stabilized zirconia (CZO–CSZ) eutectic substrates by combining dip-coating and laser floating zone (LFZ) techniques. Dip-coating provides a homogeneous and controllable precursor layer on the surface of the CZO–CSZ substrate, while LFZ processing enables localized melting followed by directional solidification, promoting the development of oriented microstructures. These techniques are complementary, and their combination allows the fabrication of novel functional coatings. The study aims to investigate the relationship between the microstructural anisotropy induced by directional solidification and the polarization dependence of the optical emission of Nd^3+^ ions. To date, and to the best of our knowledge, no studies have reported rare-earth-doped W-TCP coatings, and the polarization dependence of their optical emission has not been explored. Microstructural and compositional analyses were performed by field emission scanning electron microscopy (FESEM) and energy-dispersive X-ray spectroscopy (EDS), while micro-Raman spectroscopy was employed to assess the degree of crystallinity and possible orientation effects. Optical emission spectroscopy under controlled excitation and detection polarization was used to examine the behavior of the ^4^F_3/2_→^4^I_9/2_ transition and ^4^F_3/2_→^4^I_11/2_ transitions of Nd^3+^ ions. The correlation between vibrational and optical features allowed the origin of the observed anisotropy to be determined, demonstrating that the LFZ technique enables control over the optical polarization response of bioceramic coatings, with potential interest for photonic and biomedical applications.

## 2. Materials and Methods

Both dip-coating and laser floating zone (LFZ) techniques were combined to fabricate the neodymium-doped W-TCP coating. First, a ceramic suspension was prepared using ethanol (44.49 wt%), Beycostat C213 (0.42 wt%), polyvinyl butyral, PVB, (12.72 wt%), and 42.37 wt% of a powder mixture consisting of wollastonite (Aldrich, St. Louis, MO, USA, 99%) and tricalcium phosphate (Carlo Erba Reagenti, Cornaredo, Italy, analytical grade) in the eutectic composition of 80–20 mol%, respectively, together with 1 wt% Nd_2_O_3_ (Aldrich, 99%).

Next, the W-TCP:Nd coating was fabricated in two stages. First, CZO-CSZ eutectic rods previously prepared by the LFZ technique were dip-coated in the ceramic precursor suspension. The rods were dipped and withdrawn ten times at a constant rate of 3 mm/s to control the coating thickness and subsequently sintered at 1200 °C for 12 h to densify the layer and improve adhesion. In the second stage, directional solidification was carried out using the LFZ technique. The fundamentals of the LFZ process can be found elsewhere [25,26]. During LFZ processing, a continuous-wave CO_2_ laser was used with a power in the range of 40–50 W, adjusted to achieve surface melting of the coating to promote surface cladding without alloying with the CZO-CSZ core [27]. The laser beam, 5 mm in diameter, was focused to provide a 2 mm length molten zone. Processing was carried out under ambient air atmosphere. The thermal gradient during directional solidification was indirectly controlled by means of the laser heating and the imposed pulling rate. In this configuration, the combination of laser power and growth rate determines the solidification conditions and resulting microstructure, as widely reported for LFZ-processed eutectic systems [25]. As reported in previous works, the growth rate in this bioceramic eutectic system determines whether crystals, glass-ceramics or glasses are formed [28,29,30]. In this study, a growth rate of 100 mm/h was selected to ensure the fabrication of a glass-ceramic coating. Samples were cut and polished for microstructural and optical characterization. After polishing, the samples were annealed at 750 °C for 12 h to relieve residual stresses that may have been induced during sample preparation.

Morphology and semiquantitative composition characterization were performed on both transversal and longitudinal sections using field-emission scanning electron microscopy (FESEM, model Carl Zeiss MERLIN (Carl Zeiss AG, Oberkochen, Germany)) equipped with an energy dispersive X-ray spectroscopy (EDS) detector.

Luminescence characterization was carried out at room temperature using a home-built microscope, with the sample excited at 800 nm by a single-mode fiber-coupled diode. The backscattered light was delivered to a spectrometer (SR303i-B, Andor, Belfast, Northern Ireland) equipped with a thermoelectrically cooled CCD detector (Newton 920, Andor). The spectral resolution of the spectrometer under the experimental conditions used was 0.13 nm. Wavelength calibration was performed using the manufacturer’s calibration procedure, ensuring an accuracy better than the spectral resolution. Peak positions were determined directly from the maxima of the emission spectra, as the Stark components were well resolved. Two polarizers were placed in the optical setup to evaluate the dependence of the luminescence on polarization. The first polarizer, Pex, was used to set the excitation laser polarization parallel (∥) or perpendicular (⊥) to the growth direction of the samples. The excitation polarization was defined by a linear polarizer placed immediately before the sample. It was checked that the focusing objective did not significantly alter the linear polarization state under the experimental conditions used. Therefore, the polarization state at the sample plane was well defined for the purposes of relative polarization-dependent measurements. The second polarizer, Pem, was used to analyze the luminescence as a function of the detection polarization angle, which was rotated from 0° to 90° clockwise with respect to the excitation polarization. In this work, the convention Pem 00 and Pem 90 denotes detection polarization parallel and perpendicular to the excitation source, respectively.

Micro-Raman characterization was performed by using a confocal microscope coupled to a WITec Alpha 300 system (WITec GmbH, Ulm, Germany). A continuous-wave (cw) laser with emission at 488 nm was used to excite the sample.

For each measurement technique, at least three independent spectra were recorded at different locations of the longitudinal and transversal sections to confirm the reproducibility of the observed features.

## 3. Results and Discussion

### 3.1. Microstructural Characterization

**Figure 1:**
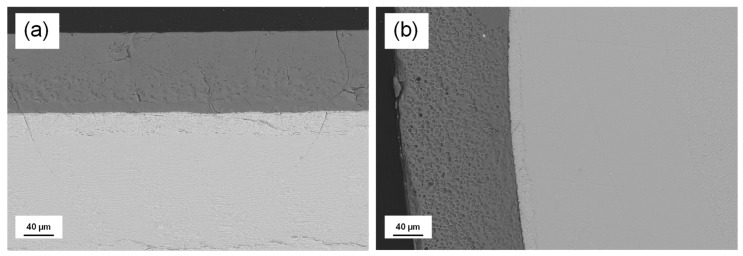
Scanning Electron micrographs of the W-TCP:Nd coating (dark contrast) cladded onto the CZO-CSZ eutectic rod (clear contrast). Image (**a**) shows a detailed view of the coatings in longitudinal section, and (**b**) shows the transversal section.

A detailed study of the microstructural characterization of the coating fabrication can be found in a previous publication [27]. In summary, the CZO-CSZ eutectic composite was first produced in cylindrical shape using the LFZ technique at a growth rate of 200 mm/h. This eutectic composite consists of two cubic phases, solidified in a lamellar distribution. Figure 1 shows SEM micrographs of the longitudinal (a) and cross-section (b) views of the W-TCP:Nd coating cladded onto the CZO-CSZ core. The clear contrast represents the CZO-CSZ core, while the W-TCP:Nd coating is represented by the dark contrast. Both micrographs showed a clean interface, confirming that the low laser power used to melt the coating minimized the dilution of the bioceramic into the eutectic core. Table 1 shows the semiquantitative compositional analysis performed on the layer, confirming a composition close to the theoretical values, also included in the table.

### 3.2. Optical Characterization

**Figure 2:**
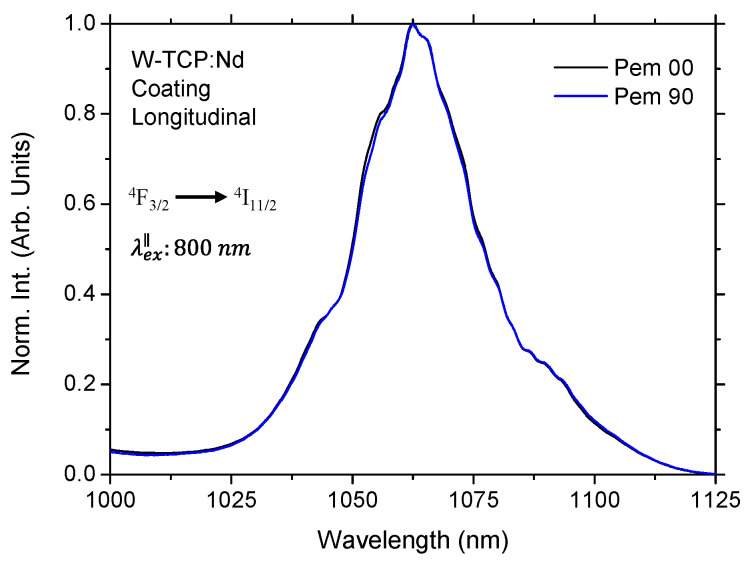
Normalized fluorescence spectra of the ^4^F_3/2_→^4^I_11/2_ laser transition measured at room temperature for the longitudinal section of the W-TCP:Nd coating by exciting the sample in parallel polarization.

The most important emission bands for the Nd^3+^ ions are those obtained by resonantly exciting the sample within the ^4^I_9/2_→^4^F_5/2,_^2^H_9/2_ absorption band. The laser transition corresponding to the ^4^F_3/2_→^4^I_11/2_ manifold is commonly studied because of its potential applications in the field of infrared optical amplification [23,24]. In addition, the features of the fluorescence spectra associated with this transition have also been studied for the evaluation of the local structure surrounding the Nd^3+^ ions and the covalency of the Nd-O bond in glass matrices [31]. However, the 4f electrons of Nd^3+^ are strongly shielded by the outer 5s and 5p orbitals, so the energies of the ^4^F_3/2_ and ^4^I_11/2_ levels are weakly perturbed by the crystal field. As a result, the emission wavelength placed at around 1060 nm is considered as a host-insensitive emission line [24]. Furthermore, this transition is relatively isotropic, showing little dependence on polarization, as shown in Figure 2, in which the steady-state emission in the longitudinal section of the W-TCP:Nd coating for polarizations parallel (Pem 00) and perpendicular (Pem 90) to the excitation source showed similar characteristics. The same behavior was observed for the transversal section of the coating under perpendicular excitation.

**Figure 3:**
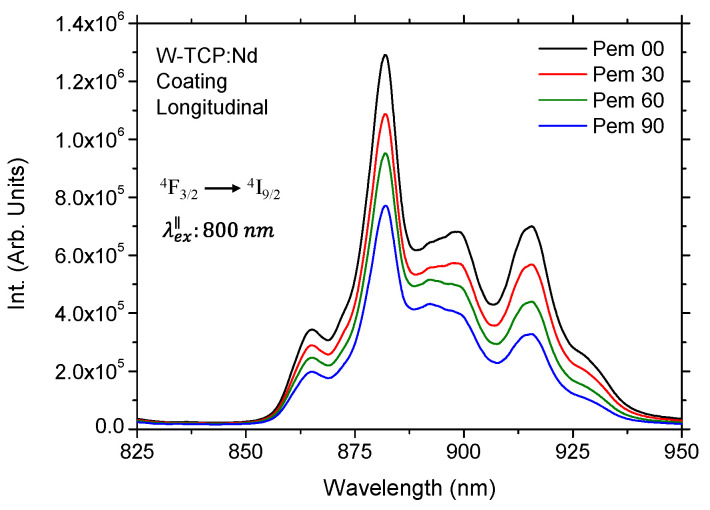
Emission spectra of the longitudinal section of the coating for emission polarization placed at 0, 30, 60 and 90 degrees to the excitation laser source.

**Figure 4:**
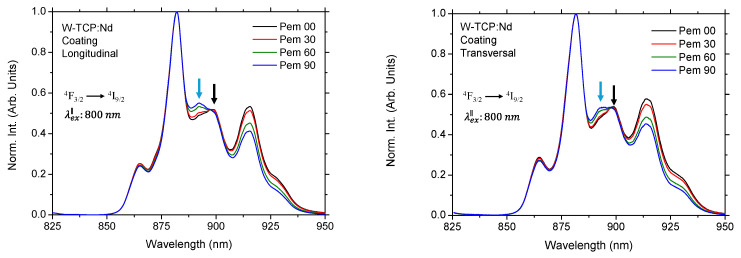
Normalized emission spectra of the longitudinal (**left**) and transversal section (**right**) of the coating for emission polarization placed at 0, 30, 60 and 90 degrees to the excitation laser source. Arrows point out the apparent blue-shift of the overall peak maximum.

In contrast, the emission band placed at 870 nm corresponding to the ^4^F_3/2_→^4^I_9/2_ transition exhibits strong Stark splitting due to the crystal field and is highly dependent on both the symmetry and the host matrix [24]. Consequently, the characteristics of the Stark sublevels are host-sensitive. In addition, the transition probabilities depend on the orientation of the light’s electric field vector, causing both the intensity and the spectral position of the Stark sublevels to be polarization-dependent. This behavior was clearly observed in the emission properties of the coating in both longitudinal and transversal sections under both excitation configurations. As an example, Figure 3 shows the emission spectra of the longitudinal section for detection polarizations set at 0°, 30°, 60° and 90° relative to the excitation laser, which in this case was aligned parallel to the growth direction. The corresponding normalized spectra are presented in Figure 4 for both longitudinal and transversal sections, as normalization enhances the contrast among the Stark components, thereby amplifying relative changes.

Regarding the incorporation of Nd^3+^ ions in the W-TCP glass-ceramic coating, Nd^3+^ is expected to substitute preferentially for Ca^2+^ sites due to their comparable ionic radii, with local charge-compensation mechanisms depending on the surrounding composition [24]. In wollastonite-rich regions, the lower local symmetry and the presence of distorted Ca-O polyhedra can lead to stronger crystal-field perturbations, favoring enhanced Stark splitting [23]. In contrast, TCP-rich regions provide different coordination environments and crystal-field strengths, which may modify the relative energy separation and intensity distribution of the Stark components [23]. As a result, the polarization-dependent redistribution of Stark components observed in the emission spectra can be explained by Nd^3+^ ions experiencing distinct local environments associated with the anisotropic microstructure induced by directional solidification.

It can be observed in Figure 3 that the emission intensity reached its maximum at the 882 nm component. Furthermore, as the detection polarization was rotated, the overall emission decreased, being highest for parallel polarization and lowest for perpendicular polarization. This intensity evolution was accompanied by changes in the spectral features of the Stark components, as shown in Figure 4. Each transition of the Nd^3+^ emission in the ^4^F_3/2_→^4^I_9/2_ manifold is governed by different electric dipole selection rules that depend on the orientation of the light’s electric field vector relative to the anisotropic crystalline phases resulting from directional solidification. The decrease in the intensity arose from a combination of lower probability of electric-dipole transition when the emission polarization was perpendicular to the optical axis, and a slight polarization-induced spectral shift. Therefore, parallel polarization (Pem 00) enhanced the intensity of parallel-polarized Stark transitions, corresponding to the components placed at 865 nm, 898.24 nm and 915 nm, whereas perpendicular polarization (Pem 90) suppressed these parallel transitions and enhanced selectively perpendicular-polarized transitions, whose main component was centered at 892.33 nm. The resulting apparent blue-shift in the overall peak maximum from 898.24 nm to 892.33 nm, pointed out by arrows in Figure 4, which was observed as for longitudinal as for transversal sections, was caused by the strong suppression of the longer-wavelength Stark component and the corresponding selective enhancement of a distinct shorter-wavelength component. Similar, although smaller than 1 nm, shifts were observed for the components near 865 nm and 915 nm. By defining the polarization anisotropy ratio as A = I_∥_/I_⊥_ for the dominant Stark component corresponding to the maximum emission intensity, values of A = 1.67 and A = 1.42 were obtained for the longitudinal and transversal sections, respectively. A comparable polarization-dependent behavior was observed under perpendicular excitation, with anisotropy ratios of the same order of magnitude.

**Figure 5:**
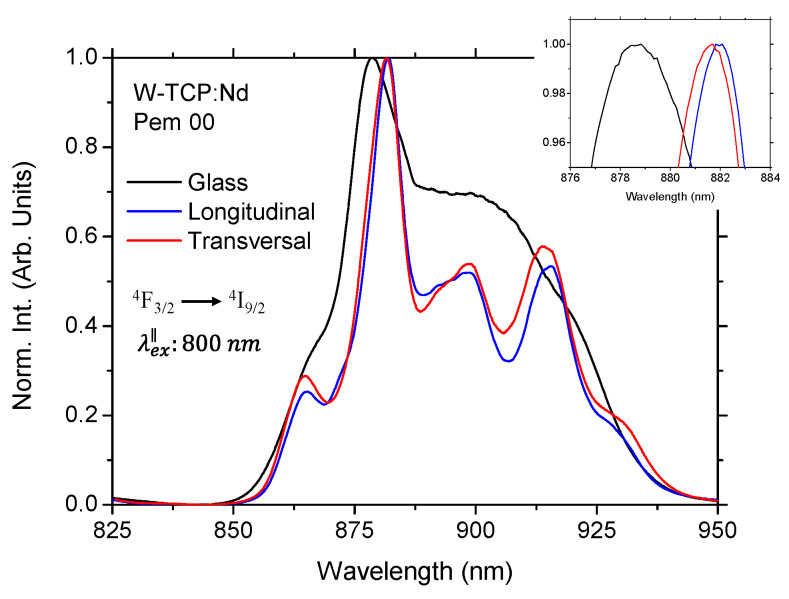
Normalized emission spectra in parallel polarization for the longitudinal and transversal sections of the W-TCP:Nd coating and W-TCP glass. The inset shows in detail the position of the highest intensity components.

Finally, a comparison of the emission spectra for both longitudinal and transversal sections of the W-TCP:Nd coating and a W-TCP:Nd glass sample was performed. As an example, Figure 5 shows the normalized emission spectra under parallel detection polarization for the 3 samples. It can be observed that the crystalline nature of the coating was clearly revealed, since the splitting of the Stark components resulted in a more complex emission spectrum [23,24]. In addition, comparison between the glass and the coating showed a 3 nm shift in the peak position of the highest-intensity component. Moreover, a spectral shift was also observed between the longitudinal and transversal sections, with the maximum intensity component located at 881.6 nm and 882.0 nm, respectively. These shifts are shown in more detail in the inset of Figure 5. In addition, it was found significant spectral differences in the Stark components of both sections. The same characteristics were observed for the other polarization configurations as for the emission as for the excitation polarization.

### 3.3. Micro-Raman Characterization

**Figure 6:**
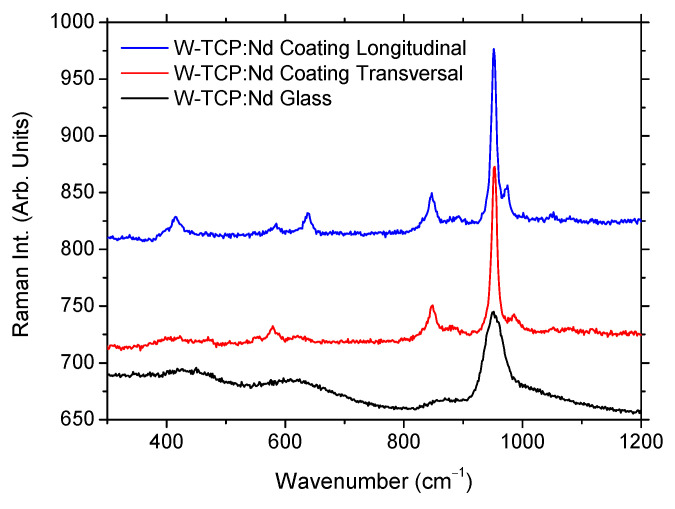
Raman spectra of the W-TCP:Nd coating and W-TCP:Nd eutectic glass.

To investigate the crystalline nature of the coating, µ-Raman characterization was carried out and compared with the W-TC:Nd glass. In addition, to clarify the observations described in the luminescence study for the longitudinal and transversal sections of the coating, Raman characterization was also performed in these sections. Figure 6 shows Raman spectra of the glass and the two coating sections in the wavenumber region 300–1200 cm^−1^. The position of peaks and bands is shown in Table 2. It can be observed that the glass sample showed a simple Raman spectra made up of few broad bands, characteristics of an amorphous silicate structure [32,33]. The bands, centered at approximately 430 cm^−1^, 623 cm^−1^, 863 cm^−1^, and 950 cm^−1^, are broad and relatively low in intensity, consistent with disordered glassy materials lacking long-range structural order. In contrast, the coating spectra showed narrow bands with higher relative intensity at similar positions to those of the glass, together with additional sharp peaks and broad bands, suggesting higher degree of structural ordering or differences in bonding environments, possibly due to orientation effects and/or the presence of crystalline phases within the coating [32,33].

**Figure 7:**
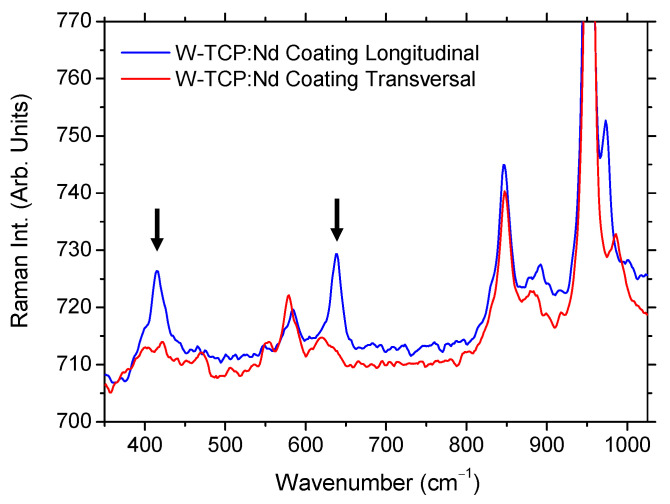
Comparison of Raman spectra of the longitudinal and transversal sections of the W-TCP:Nd coating. Arrows indicate vibrational modes characteristic of the longitudinal coating.

A more detailed comparison of the vibrational differences between the longitudinal and the transversal sections is shown in Figure 7, which reflects the anisotropic microstructure induced during directional solidification. The longitudinal section exhibited sharper and more intense Raman modes, particularly the peaks at 415.2 cm^−1^ and 639.5 cm^−1^. The band at 415.2 cm^−1^ was absent in the transversal section, indicating that this vibrational mode was associated with the crystalline phases oriented along the LFZ growth direction. Likewise, the 639.5 cm^−1^ mode underwent a clear red shift and a decrease in intensity in the transversal section, suggesting modifications in the local bonding environment, most likely due to changes in Si–O–Si and Si–O–Ca linkages induced by anisotropic stress fields [34].

In the transversal section, a small broad band centered at around 552.2 cm^−1^ appeared, together with an enhancement of the peak at 578.8 cm^−1^, which became more intense than in the longitudinal section. This behavior is characteristic of a less ordered or differently oriented polyhedral network, consistent with the expected microstructural differences perpendicular to the solidification front [34,35]. In addition, the band at 891.4 cm^−1^ underwent a red shift whereas the mode at 972.7 cm^−1^ showed a blue shift. These opposing shifts in the peak positions suggest different crystallographic orientations and anisotropic stress along the growth direction induced during the directional solidification process.

The evolution of peak positions, intensities and bandwidths between both longitudinal and transversal sections evidences an orientation-dependent microstructure, confirming both the glass-ceramic nature of the coating and the anisotropic properties of its crystalline phases. These Raman results are consistent with the polarization-dependent luminescence observations, further supporting the conclusion that directional solidification induces optical and structural anisotropy in the W-TCP:Nd coating.

The polarization-dependent emission demonstrated here opens the possibility of developing passive, contactless optical tracking and sensing strategies in bioactive implants, where orientation, microstructural integrity, or stress-induced changes could be monitored through polarization-resolved optical readout.

## 4. Conclusions

- Neodymium-doped bioactive W-TCP coatings were successfully produced by the combined use of dip-coating and LFZ techniques, resulting in homogeneous and well-adhered glass-ceramic layers on the CZO-CSZ eutectic core.
- Raman spectroscopy revealed new vibrational modes and distinct peak shifts between longitudinal and transversal sections. The longitudinal section exhibited sharper and more intense peaks, whereas the transversal section showed broader features and red-shifted bands, indicating anisotropic stress and crystallographic orientation along the growth direction.
- The ^4^F_3/2_→^4^I_9/2_ transition presented Stark splitting and strong polarization dependence in both emission intensity and spectral position, while the ^4^F_3/2_→^4^I_11/2_ transition remained nearly isotropic.
- The optical anisotropy observed in the luminescence is directly correlated with the microstructural anisotropy induced by the directional solidification process, confirming the role of the LFZ technique in controlling orientation and optical response.
- The Nd-doped W-TCP coating combines bioactive ceramic behavior with controlled optical anisotropy. Beyond the fundamental optical–structural relationship, the results highlight its potential as multifunctional bioactive materials with integrated polarization-sensitive optical functionality for implant-related sensing and tracking applications.

## Figures and Tables

**Table 1:** Composition of the Neodymium doped W-TCP eutectic coating.

at.%	O	Ca	P	Si	Nd
Coating mean composition	59.28	23.50	7.40	9.77	0.05
Theoretical	60.60	21.21	6.06	12.12	

**Table 2:** Raman frequency modes for the W-TCP:Nd eutectic glass and both longitudinal and transversal sections of the W-TCP:Nd coating.

Sample	Frequency (cm^−1^)
Glass	430, 623, 863, 950
Coating: Longitudinal	415.8, 585.0, 638.8, 846.4, 891.4, 951.9, 972.7, 1050.2, 1097.5
Coating: Transversal	472.5, 553.8, 578.8, 629.3, 847.4, 881.6, 953.2, 985.4, 1052.9, 1082

## Data Availability

The original contributions presented in this study are included in the article. Further inquiries can be directed to the corresponding author.
